# Jewel Scarabs (*Chrysina* sp.) in Honduras: Key Species for Cloud Forest Conservation Monitoring?

**DOI:** 10.1673/031.013.2101

**Published:** 2013-03-18

**Authors:** M. Jocque, M.P.M. Vanhove, T.J. Creedy, O. Burdekin, J.M. Nuñez-Miño, J. Casteels

**Affiliations:** 1 BINCO vzw, Rijmenamsesteenweg 189, 3150 Haacht, Belgium; 2 Operation Wallacea, Hope House, Old Bolingbroke, Lincolnshire, United Kingdom; 3 Institute of Biodiversity and Ecosystem, Bulgarian Academy of Sciences, 2 Yurii Gagarin Street, 1113 Sofia, Bulgaria; 4 Laboratory of Biodiversity and Evolutionary Genomics, Biology Department, KU Leuven, Ch. Deberiotstraat 32, 3000 Leuven, Belgium; 5 Hope Entomological Collections, Oxford University Museum of Natural History, Parks Road, Oxford OXI 3PW, United Kingdom; 6 Department of Zoology, University of Oxford, South Parks Road, Oxford OX1 3PS, United Kingdom; 7 Laboratory of Aquatic Ecology, Evolution and Conservation, Biology Department, KU Leuven, Ch. Deberiotstraat 32, 3000 Leuven, Belgium

**Keywords:** biodiversity, checklist, conservation, elevation, jewel scarab

## Abstract

Jewel scarabs, beetles in the genus *Chrysina* Kirby (Coleoptera: Rutelinae: Scarabaeidae), receive their name from the bright, often gold, green elytra that reflect light like a precious stone. Jewel scarabs are commonly observed at light traps in Mesoamerican cloud forests, and their association with mountain forests makes them potentially interesting candidates for cloud forest conservation monitoring. The absence of survey protocols and identification tools, and the little ecological information available are barriers. In the present study, collection of *Chrysina* species assembled during biodiversity surveys by Operation Wallacea in Cusuco National Park (CNP), Honduras, were studied. The aim of this overview is to provide an easy to use identification tool for in the field, hopefully stimulating data collection on these beetles. Based on the data associated with the collection localities, elevation distribution of the species in the park was analyzed. The limited data points available were complemented with potential distribution areas generated with distribution models based on climate and elevation data. This study is aimed at initializing the development of a survey protocol for *Chrysina* species that can be used in cloud forest conservation monitoring throughout Central America. A list of *Chrysina* species recorded from Honduras so far is provided.

The six identified and one unidentified species recorded from CNP are easy to identify in the field based on color and straightforward morphological characteristics. Literature research revealed ten species currently recorded from Honduras. This low species richness in comparison with surrounding Central American countries indicates the poor knowledge of this genus in Honduras. *Chrysina* species richness in CNP increases with elevation, thereby making the genus one of a few groups of organisms where this correlation is observed, and rendering it a suitable invertebrate representative for cloud forest habitats in Central America.

## Introduction

Mountain forests, and cloud forests in particular, in Central America remained largely undisturbed by humans for a considerably longer time than lowland forests, mostly due to their limited accessibility. However, an increasing demographic pressure is currently seriously affecting this habitat, to the extent that cloud forests are high on the list of the worlds’ most threatened ecosystems (Aldrich et al. 1997; Bruijnzeel and Hamilton 2000). As a consequence, cloud forests in Central America persist mainly as small, scattered fragments (Cayuela et al. 2006). A growing awareness of deforestation and recognition that natural mountain areas play an essential role in providing drinking water in Honduras led to the Cloud Forest Decree in 1987 (Decreto legislativo 87–87), which declared all forests above an elevation of 1800 m a.s.l. as protected areas. The resulting creation of national parks was a step towards the protection of these unique habitats; however, it offered no guarantee of effective conservation (Martin and Blackburn 2009). Many of these areas are “paper parks,” with no implementation of their protected status and no forest guards to supervise human activities. These regions are threatened by illegal logging, mostly for cattle pasture and coffee farms. As an example, Cusuco National Park (CNP) in northern Honduras suffered an annual deforestation rate of approximately 0.2% between 1987 and 2000 (House et al. 2005). Increased conservation efforts are required to protect this habitat, and key taxonomic groups that can be used in cloud forest monitoring should be identified. There are several vertebrate groups used in monitoring programs in Central American cloud forests, while the use of invertebrates is rare. This lack of invertebrate usage is partly because most groups are understudied, and survey protocols and identification tools are lacking.

*Chrysina* spp. Kirby (Coleoptera: Rutelinae: Scarabaeidae) are large, bright-green to silvergold colored beetles, appropriately named jewel scarabs. The charismatic presence and remarkable colors of *Chrysina* species have made them desired possessions for beetle collectors. These insects, in particular *C. gloriosa* (LeConte), are often used as study organisms to look into the morphogenesis and optical characteristics of beetle exoskeletons (e.g., Brady and Cummings 2010). Insights from these studies have applications in the development of tuneable micro-mirrors or microlens arrays (Agez et al. 2011) and chirooptical devices (Sharma et al. 2011). At present, there are 96 described species of *Chrysina* (Hawks 2006), distributed from Mexico to Ecuador. Little is known about their ecology, but they are generally found in primary pine, juniper, and pine-oak forests, up to an elevation of 3800 m a.s.l. Adults feed on foliage and larvae on rotting logs of various tree species. Their association with mountain forests links these animals with pristine, but highly threatened, habitats in Central America.

On light trapping sessions in the cloud forest of CNP, *Chrysina* species are a common occurrence. To facilitate ongoing biodiversity surveys, an illustrated overview of *Chrysina* species from CNP is presented here. Furthermore, an identification guide and accompanying species accounts are provided for use in the field, and are based on easy to identify morphological characteristics and color. To illustrate the potential of *Chrysina* species as focal group for cloud forest biodiversity conservation monitoring in Mesoamerican mountainous forests, the potential distribution of *Chrysina* in the park was modelled based on climate (precipitation and temperature) and elevation associated with collection specimens housed in the Oxford University Museum of Natural History (OUMNH). An overview of *Chrysina* species recorded from Honduras is provided. The goal of this paper is to stimulate data collection on *Chrysina* species, in CNP in particular, as well as Central America in general, and to contribute to the conservation of the remaining cloud forests.

## Materials and Methods

The data presented in the manuscript stem from *Chrysina* species in the OUMNH assembled during biodiversity surveys of the ecovolunteer-driven conservation organization Operation Wallacea in CNP during the summer field seasons (June-August) between 2006 and 2011.

### Field site

CNP is situated in northwestern Honduras (Figure 2) within the Merendon Mountain range. The core zone of the park consists of lower montane tropical rainforest (a mix of primary and secondary forest), with patches of primary cloud forest and upper montane rainforest with a maximum elevation of 2245 m.a.s.l.

Data collection was centered on seven camp locations in CNP. Location details for these camps are given in UTM (Zone 16P) GPS coordinates along with elevation (m a.s.l.): Base Camp (370040, 1713699, 1546 m), Guanales (368060, 1712000, 1270 m), Cantilles (367044, 1715388, 1828 m), Buenos Aires (373498, 1713954, 1144 m), Santo Tomas (360586, 1720884, 534 m), Cortecito (361758, 1716344, 1397 m) and Danto (363044, 1717193, 1596 m). For most specimens, individual coordinates were available. When exact coordinates of the collection locality were missing, the closest camp was used as the collection point.

### Sampling protocol

**Figure 1.**
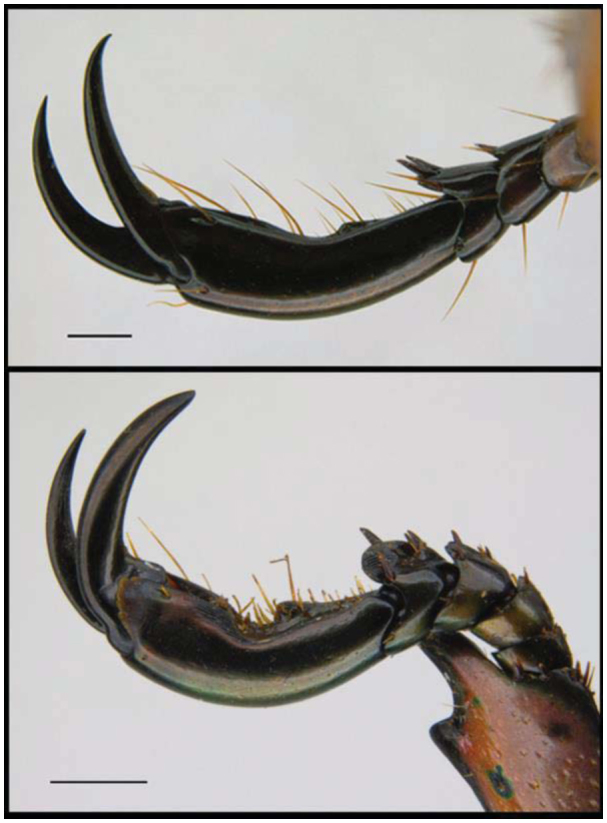
Claws of female (upper) and male (lower) *Chrysina spectabilis*. Scale represents l mm. Image: T. Creedy. High quality figures are available online.

Specimens were collected (not trapped) at UV lights, the most efficient way of collecting Rutelinae (Garcia-Lopez et al. 2011). The set up used was a Gladiator 25W Actinic light running on a 12 V car battery. Specimens were preserved in 70% ethanol and vouchers were deposited in the Hope Entomological Collections (accession numbers: OUMNH2006–082; OUMNH-2008–052; OUMNH2009–054; OUMNH-2011–066). These collection batches formed the basis of the current study. Specimens were measured from the tip of the clypeus to the end of the pygidium (Morón 1990). Additional measurements were taken from the tip of the clypeus to the end of the elytra. Pinned specimens sometimes have a slight curve in the body, and presented values may be slight underestimations of the actual size. For subsequent analyses, the maximal length (up to either elytra or pygidium) was used. Males and females were distinguished based on shape and development of the front leg tarsal claws. In males, these are more strongly developed and rather pointed inwards, compared to females (Figure 1).

### Identification and analyses

The *Chrysina* species were identified using the information presented by Hawks (2006) and Morón (1990) and species descriptions subsequently published (e.g., Rattcliffe and Jameson 1992; Curoe 1994).

Species accounts are provided to allow easy identification of the CNP *Chrysina* species in the field, presenting average sizes, color descriptions, distributions in CNP, and easy to recognize morphological characteristics from the specimens in Hope Entomological Collections (OUMNH). An identification guide for use in the field is provided.

The distribution of species richness was examined across the altitudinal gradient in CNP. Correlations were calculated in STATISTICA 64 10 (Statsoft, Inc., www.statsoft.com). Distributional data in Mesoamerica were retrieved from Hawks et al. (2006) and references therein.

The data available in the collection was from a limited number of points, and accompanying ecological data (elevation, precipitation and temperature) was used with the specimens to model expected distribution for each species in CNP. The information on the samples in decimal degrees was transferred to UTM coordinates using the spreadsheet used for batch converting lat/long coordinates to UTM (www.spatialscrawl.com/2010/10/technical-tip-converting-latlong-to-utm-coordinates-ordms-to-dd). The maximum entropy model was constructed with MaxEnt 3.3.3 (Phillips et al. 2004). With only two recorded localities for both *C. strasseni* (Ohaus) and *C*. sp1., models were not able to be built for these species. A model was developed for the remaining five species using 8–71 occurrence records (Table 1) and three environmental variables (temperature, precipitation, and elevation). Climate data (average yearly precipitation and temperature) were generated from the WorldClim database (www.worldclim.org). The WorldClim climate layers have a resolution of 1 km (Hijmans et al. 2005), so this resolution was used in the analyses. The environmental data layers were cropped to the boundary of the buffer zone of CNP. MaxEnt was run under the “auto-features” mode and default settings (Phillips and Dudik 2008). “Logistic output” format was used, as the estimated species probability of presence is easier to interpret considering the constraints imposed by environmental variables (Phillips and Dudik 2008). Default MaxEnt parameters were used with replicates generated by cross-validation. This method uses all of the data for validation, thus making better use of small data sets. To increase the heterogeneity of replicate datasets, n-1 replicates were used, in which n is the number of occurrence records, as suggested by Pearson et al. (2007), when a small number of occurrence records are available. The final model for each species was based on the mean of the replicated models. The strength of the composed models was evaluated based on the area under curve of the receiver operating characteristics analysis. Values approaching 1 are considered robust predictions of the expected occurrence of the species (Phillips et al. 2006). The contribution and value of each predictor variable was evaluated with a jackknife test of variable importance.

**Table 1.**
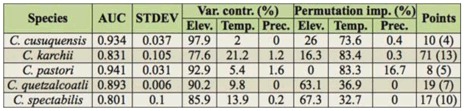
Statistics for the MaxEnt distribution models composed for five of the six *Chrysina* species in Cusuco National Park, Honduras. The final model considered is the average of the replicate models. Presented here are the Area under Curve (AUC) and standard deviation (STDEV). The variance contribution of the three predictor variables (elevation, precipitation, and temperature) and their permutation importance was evaluated with a jackknife test, and results are presented in percentages. The number of records and unique collection localities in between brackets are mentioned for each species in the last column.

### Imaging

Images were created using the PCphotomicroscopy equipment at OUMNH. The equipment consists of a Leica M165C microscope (www.leica-microsystems.com) with motorized focus and a LED5000 HDI dome light. All images are based on a focus stack of each specimen taken using Leica Application Suite and photomontaged with Helicon Focus 5.2.11 (www.heliconsoft.com). Images were edited in Adobe Photoshop CS5 (www.adobe.com) to correct color balance, improve lighting, and achieve a uniform background.

## Results

A total of 144 *Chrysina* specimens were available for study in the OUMNH collections, making this collection the second largest Chrysina collection from CNP. Seven *Chrysina* species were distinguished, and a number of remarkable color polymorphisms and variations were documented. A detailed description is presented below in the species accounts. Six species were positively identified. The seventh species could not be identified, and so is here referred to as *Chrysina* sp1. It may represent an undescribed species, material of males for use of the genitalia structure is needed to confirm the identification.

**Figure 2.**
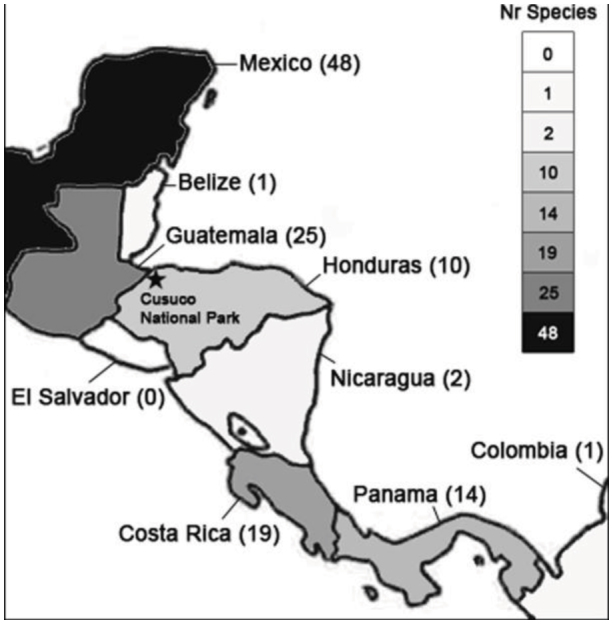
Number of *Chrysina* species recorded from the Mesoamerican countries. Data from Hawks (2006). High quality figures are available online.

At present, 10 species are known from Honduras. On a national scale, the greatest number of species is recorded from Mexico and Guatemala, followed by Costa Rica and Panama (Figure 2). The remaining Central American countries such as Honduras, Nicaragua, Belize, and El Salvador display disproportionally lower species richness.

**Figure 3.**
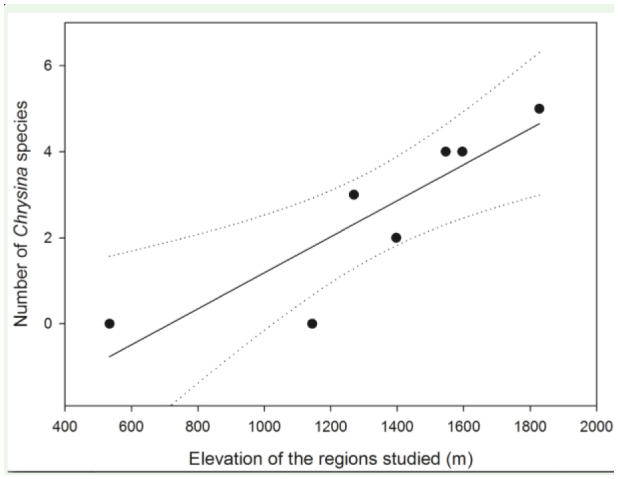
Correlation between the number of *Chrysina* species and the elevation of the sampling locations in Cusuco National Park. The dotted line represents the 95% confidence interval. High quality figures are available online.

**Figure 4.**
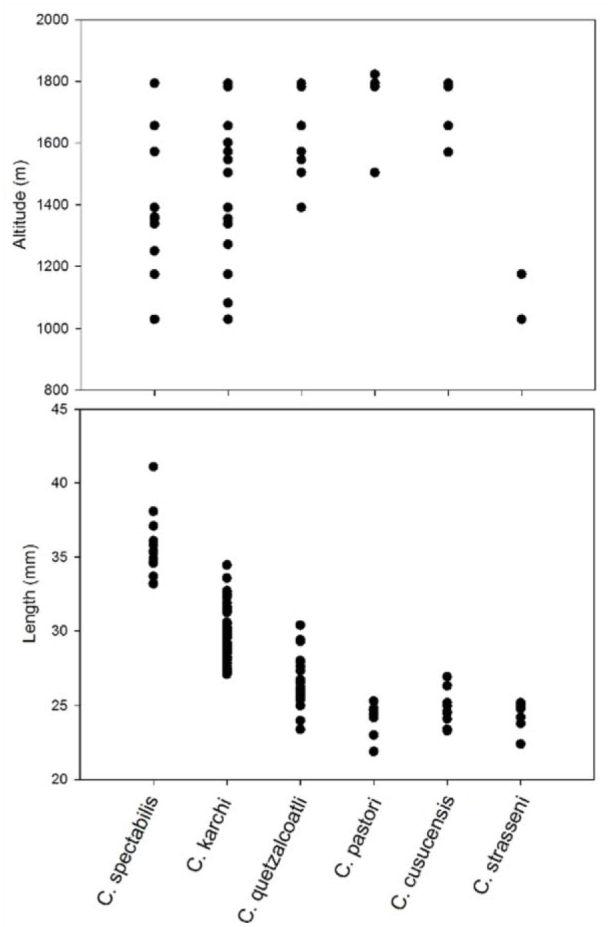
Visual representation of the elevation range and body size (measured as the maximum length) of six *Chrysina* species recorded in Cusuco. High quality figures are available online.

Species richness in CNP increased with elevation (*p* = 0.0095, R^2^ = 0.77) (Figure 3). The altitudinal range differed between the species (Figure 4) with an overall tendency to be negatively associated with body size (Figure 4). Both of the larger species (*C. spectabilis* (Ratcliffe and Jameson) and *C. karschi* (Nonfried)) were found at all elevations. Conversely, smaller congeners, except for *C. strasseni*, were restricted to higher elevations. *C. strasseni* was only recorded at Guanales, which is at a lower elevation (1130–1147 m a.s.l.). The expected distribution reflects that most species are to be found in the higher regions of the park only, with *C. strasseni*, at mid-elevation, displaying a much wider range than its congeners. *C. pastori* (Curoe) is predicted to have the most restricted distribution and appears to be limited to the highest regions of CNP, and is potentially the rarest *Chrysina* species in CNP.

**Figure 5.**
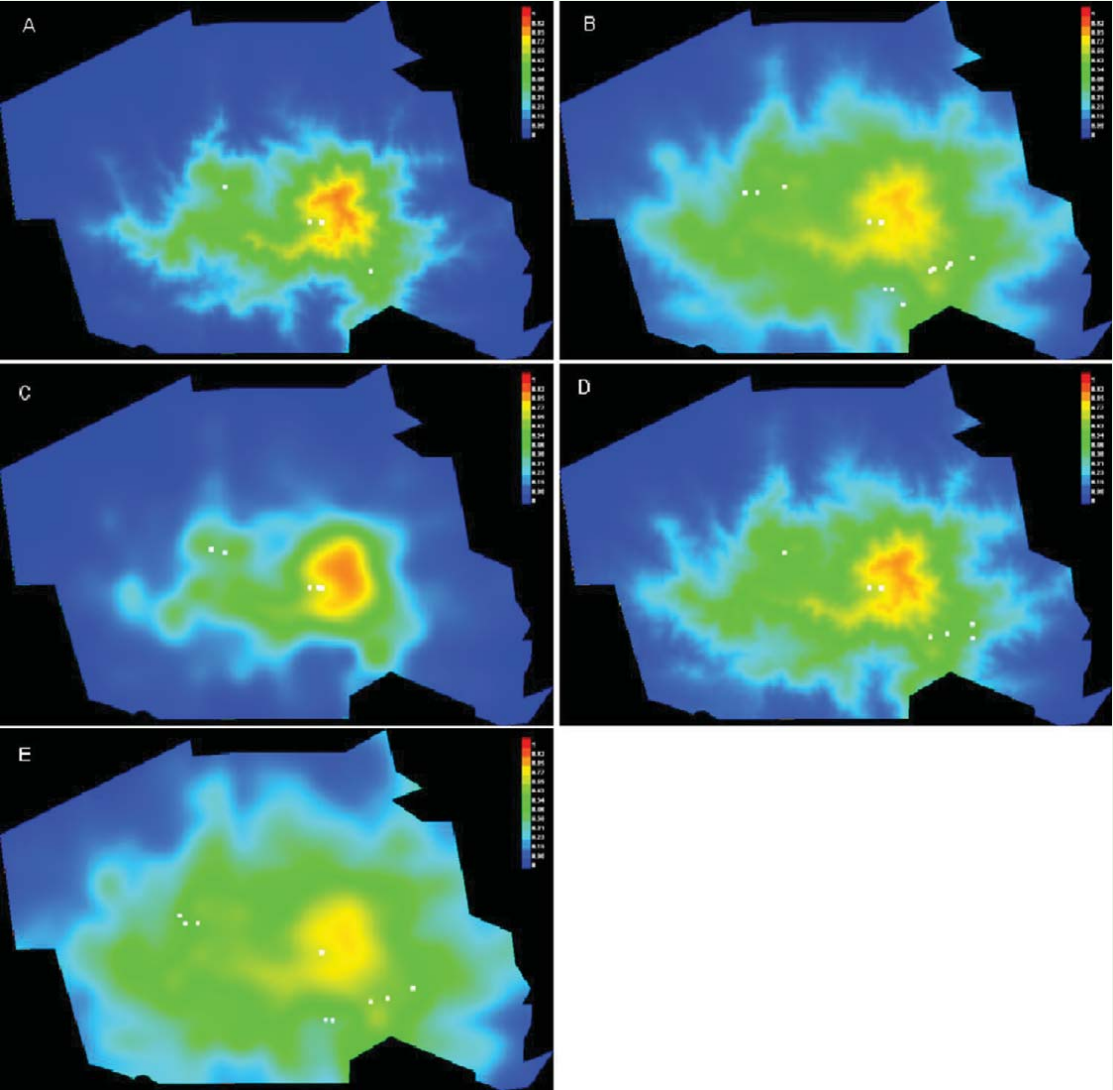
Figure presenting a prediction of the distribution of A) *Chrysina cusuquensis* B) *Chrysina karschi* C) *Chrysina pastori* D) *Chrysina quetzalcoatli* E) *Chrysina spectabilis* in Cusuco National Park (colored area) based on elevation, temperature, and precipitation. Warmer colors show areas with better predicted conditions. White dots show the collection localities. High quality figures are available online.

The potential distribution of the five modelled species further confirms the relation with altitude. The core zone of the park with elevations above 1500 m a.s.l. had higher probability values for all *Chrysina* species included in the analyses (Figure 5). The models showed a very good overall performance, presenting high area under curve values both in training and test data for *C. cusuquensis* (Curoe) and *C. pastori* (Table 1). A moderately good performance was recorded for the models of *C. karschi, C. spectabilis*, and *C. quetzalcoatli* (Morón) (Table 1). Elevation was the variable that contributed most to the predicted distribution when used in isolation, and the one that most decreased the gain when omitted. Among the two climate variables, temperature contributed most to the models.

### Field identification key to *Chrysina* species of CNP

This identification key is based on the *Chrysina* collection in the OUMNH, is applicable only to CNP, and should be used together with the presented species accounts. *Chrysina* specimens that do not fit the provided identification key should be treated as a potential new species to CNP and should be identified through an examination of the male genitalia.

**Figure 6.**
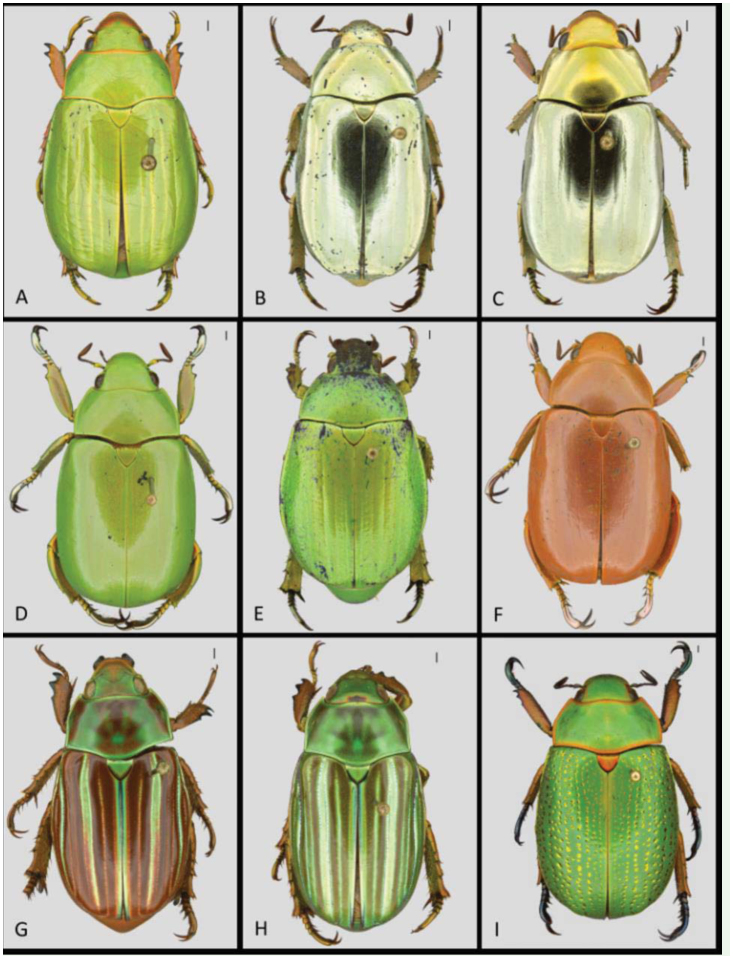
Dorsal view of *Chrysina* species in Cusuco National Park. A) *C. cusuquensis*, B) *C. pastori*, C) *C. strasseni*, D) *C. karschi*, E) *C*. sp., F) *C. karschi* rare pink morph, G) *C. quetzalcoatli*, H) *C. quetzalcoatli*, green morph, l) *C. spectabilis*. Scale (top right) indicates l mm. Images: T. Creedy. High quality figures are available online.

**Figure 7.**
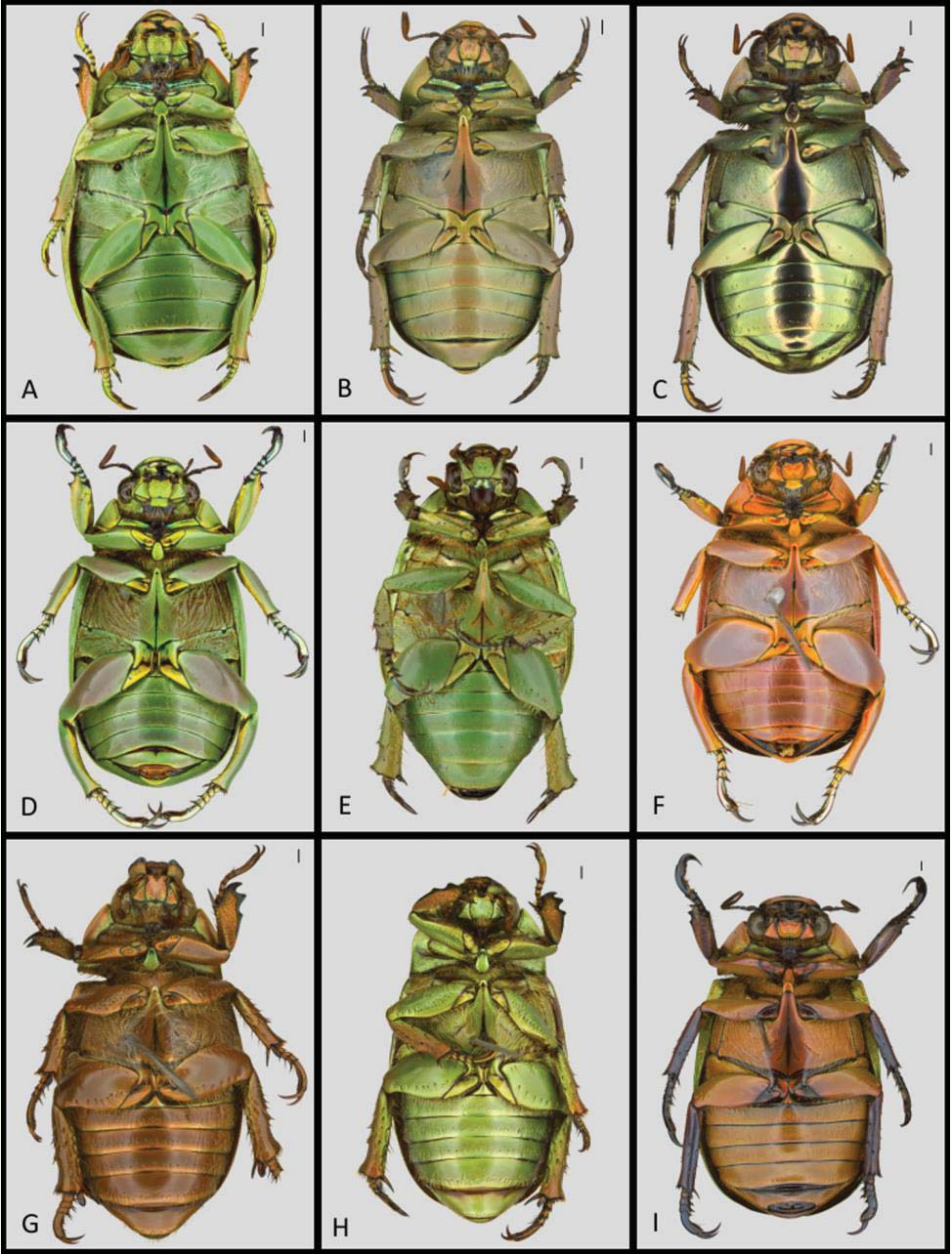
Ventral view of *Chrysina* species in Cusuco National Park. A) *C. cusuquensis*, B) *C. pastori*, C) *C. strasseni*, D) *C. karschi*, E) *C*. spl, F) *C. karschi* rare pink morph, G) *C. quetzalcoatli*, H) *C. quetzalcoatli*, green morph, l) *. spectabilis*. Scale (top right) indicates l mm. Images: T. Creedy. High quality figures are available online.

1. Dorsum (elytra and pronotum) predominantly green 2
- Dorsum (elytra and pronotum) predominantly metallic silver, pink or brown 6
2. Rows on elytra with large golden impressions; scutellum orange; large species (> 3 3 mm) (Figure 6I, 7) ***C. spectabtlts***

- Rows on elytra without large golden impressions; scutellum green; smaller species (≤ 33 mm) 3
3. Dorsum (head, pronotum, and elytra) uniformly green; at most with faint yellow margin around pronotum 4
- Dorsum (head, pronotum, and elytra) with other color besides green and with rows on elytra gold-yellow or brown-green 5
4. Rows on elytra faint, almost absent, with minute golden dots, punctures absent, hind femora proximally same width as distally with small bristles, tarsi silver metallic (Figure 6D–7D) ***C. karschi***

- Rows on elytra moderately impressed, with numerous small punctures, golden dots absent, hind femora proximally about twice as broad as distally, and dorsally with strong bristles, tarsi dark metallic brown-green (Figure 6E–7E) ***C*. sp1**
5. Pronotal margin orange, rows on elytra gold colored, depressed with punctures, third row without punctures (Figure 6–7A) *C. cusuquensis*

- Pronotal margin green, pronotum with brown blotches on a green background; rows on elytra alternating green and brown; third row with punctures (Figure 6H–7H) ***C. quetzalcoatli*** (green morph)
6. Dorsum (elytra and pronotum) metallic gold-silver 7
- Dorsum (elytra and pronotum) differently colored (striped or metallic brown pink) **8**

7. Ventrum metallic gold-silver; head and pronotum predominantly metallic gold, pygidium shiny (Figure 6C–7C) ***C. strasseni***

- Ventrum dull, pink brown; head and pronotum predominantly metallic silver, pygidium dull (Figure 6B–7B) ***C. pastori***

8. Dorsum (elytra) brown, with longitudinal green rows (Figure 6G–7G) ***C. quetzalcoatli***

- Dorsum (elytra, pronotum, head) uniformly orange-pink (Figure 6F–7F) ***C. karschi*** (rare pink morph)

### Species accounts

For all species recorded in CNP, the size of the specimen in the collection at the OUMNH is presented and compared to the size mentioned in literature. Furthermore, diagnostic characteristics are presented based on color and variation as observed on the CNP speci- mens in the OUMNH collections. These characteristics should be used together with the identification key to identify the species in the field. Also, the distribution outside as well as inside CNP is presented.

### Chrysina cusuquensis

**Size in CNP:** F (female) 26.1 ± 1.0 mm, M (male) 24.2 ± 0.7 mm, ALL 24.8 ± 1.2 (n = 10); within range of size mentioned by Curoe (1994): M 23.0–27.5mm, F 25.5–30.0mm.

**Recognition in the field:** Small size, distinguishable from all other species due to the bright yellow-green dorsal color with goldyellow slightly depressed rows and absence of punctures (Figure 6A). Anterior and lateral edges of clypeus bronze-orange, centrally bright yellow-green. Laterodorsal side of legs (femora and tibiae) bright yellow-green, lateroventral side bronze-orange. Claws greengold (Figure 7A).

**Color variation:** Most of the variation is observed in the coloration of the dorsal sides of the legs (femora and tibiae), with varying dominance of the bronze-orange with hints of pink over the yellow-green. Leg color ranges from completely bronze-orange to nearcompletely yellow-green.

**Distribution in CNP:** Altitudinal range 1570–1794 m a.s.l. (Figure 4), found in Cantiles, Danto, and Base Camp.

**Distribution:** Endemic to Cortés province of Honduras (Thomas et al. 2009).

**Remarks:** This species was described from CNP, the original type series consisting of 81 individuals, collected by D. Curoe in Cantiles from 1 to 10 July 1994 at an elevation of 1840 m a.s.l. (Curoe 1994). Identification based on male genitalia can be made through Curoe (1994).

### Chrysina karschi

**Size in CNP:** F 30.6 ± 1.8 mm, M 29.2 ± 1.5 mm, ALL 29.1 ± 1.8 mm (n = 71); on average smaller than mentioned by Morón (1990): 33 mm.

**Recognition in the field:** Distinguishable from all other species in CNP due to the uniform bright-green dorsal color, with a smooth surface without punctures and only faintly developed non-depressed golden rows (Figure 6D). Ventral color yellow-green with purple shine. Ventral side legs green with purple and golden shine in the femora and tibiae, tarsi metallic silver with greenish shine (Figure 7D). Clypeus green, smooth, with fine dense punctures. Postero-apical process of male metafemora acute (Figure 7D).

**Color variation:** Substantial color variation is observed in CNP material. The most remarkable color morphs are the individuals with an orange-brown clypeus and individuals that are completely pink (Figure 6F–7F). The color of femora and tibiae varies from a very faint yellow-gold to an intense gold.

**Distribution in CNP:** Altitudinal range 1030–1794 m a.s.l. (Figure 4), recorded from Guanales, Base Camp, Cantiles, Cortecito, and Danto.

**Distribution:** Guatemala (Young 1999), Cortés province of Honduras (Morón 1990; Thomas et al. 2007a).

**Remarks:** The fine punctuations on the clypeus of specimens in CNP are different from the holotype deposited in the ZMHU (Morón 1990), but all other characteristics, including genital structure, are similar. Identification based on male genitalia can be made through Morón (1990).

### Chrysina pastori

**Size in CNP:** F 24.4 ± 0.9 mm, M 23.7 ± 1.2 mm, ALL 24.0 ± 1.2 (n = 9); in the same range as mentioned by Curoe (1994): M 23.5– 26.5 mm, F 23.5–26.5 mm.

**Recognition in the field:** Distinguishable from all other species in CNP based on the metallic silver-gold dorsum (Figure 6B) com- bined with the shiny pink-brown ventrum (Figure 7B). Elytra smooth, rows barely visible, not depressed. Clypeus and sometimes small rim around the eyes pink-brown, nonreflecting due to fine punctures. Dorsal side of legs (femora, tibiae, and claws) shiny pinkbrown. Tarsi metallic silver-gold with a green shine. Abdominal sternites yellow-green (Figure 7B). Pygidium green with pink-brown shine, rugose.

**Color variation:** Most variation is observed in the color of the pygidium, varying from green with pink-brown shine to almost completely pink-brown with green shine. The collection contained one female with a more silvery dorsal side and a hint of green. The central part of the clypeus was shiny.

**Distribution in CNP:** Altitudinal range 1504–1823 m a.s.l., recorded from Cantiles, Cortecito, and Danto.

**Distribution:** Endemic to Cortés province of Honduras (Thomas et al. 2007b).

**Remarks:** The species patronym honors ecologist Rodrigo Pastor Fasquelle and the Ecological Foundation that bears his name for his efforts in establishing protected areas in Honduras, among them being CNP (Curoe 1994). Identification based on male genitalia can be made through Curoe (1994).

### Chrysina quetzalcoatli

**Size in CNP:** F 26.8 ± 1.6 mm, M 25.6 ± 1.4 mm, ALL 26.4 ± 1.6 mm (n = 19); in the size range mentioned by Morón (1990): 26–30 mm.

**Recognition in the field:** The only jewel scarab in CNP with longitudinal yellowgolden green lines alternating with brown lines on the elytra (Figure 6G). First rows vague, little depressed, which is the key external characteristic to distinguish this species from its Mexican congener *C. adelaida* (Hope). Scutellum yellow-gold green. All legs (femora, tibiae, tarsi, and claws) dorsally metallic bronze-brown. Clypeus metallic bronzebrown, posteriorly with a small circular depression coarse due to fine punctures, remaining part of the clypeus smooth. Pronotum with brown blotches on green background. Ventral side bronze-brown (Figure 7G), all legs (femora, tibiae, tarsi, and claws) ventrally bronze-brown, sometimes with slight green iridescence.

**Color variation:** One green color morph in the collections with the dorsal brown lines on the elytra faint and almost green (Figure 6H–7H), femora dorsally metallic green. **Distribution in CNP:** Altitudinal range 1391–1741 m a.s.l. (Figure 3), recorded from Base Camp, Cantiles, Danto.

**Distribution:** South Mexico, Guatemala, and Honduras (Thomas et al. 2006a).

**Remarks:** According to Morón (1990), this species is known from oak and pine forests between 1300–2400 m a.s.l. Identification based on male genitalia can be made through Morón (1990).

### Chrysina spectabilis

**Size in CNP:** F 39.1 ± 2.8 mm, M 35.2 ± 1.2 mm, ALL 35.4 ± 1.8 mm (n = 17); smaller than the size mentioned by Ratcliffe et al. (1992): 41.5 mm.

**Recognition in the field:** The largest *Chrysina* species in CNP, it can easily be distinguished by rows with large golden impressions on bright-green elytra (Figure 6I). Lateral rows with largest golden impressions, gradually becoming smaller towards the central line. Scutellum metallic orange. Femora and tibiae as well as first digits of tarsi dorsally metallic orange-brown. Claws and last tarsi metallic black with a faint green shine. Femora ventrally metallic orange-brown and tibiae of all legs ventrally metallic dark bordeauxviolet (Figure 7I).

**Color variation:** The tibiae vary from metallic orange-brown to metallic orange-yellow.

**Distribution in CNP:** Altitudinal range 1030–1794 m a.s.l. (Figure 4), recorded from Base Camp, Guanales, Cantiles, Cortecito, and Danto.

**Distribution globally:** Endemic to Honduras (Thomas et al. 2006b).

**Remarks:** Identification based on male genitalia can be made through Ratcliffe et al. (1992).

### Chrysina strasseni

**Size in CNP:** F 24.7 ± 0.6 mm, M 22.4 mm, ALL 24. ± 0.9 mm (n = 8); smaller than the size mentioned by Morón (1990): 26 mm. 

**Recognition in the field:** Together with *C. pastori* this species is distinguishable from all other *Chrysina* in CNP by the gold-silver dorsal color (Figure 6C). *C. strasseni* can be differentiated from the last by having the ventrum gold-silver (Figure 7C) as compared to pink-brown in *C. pastori*. Elytral rows faintly depressed with fine punctures. Legs (femora, tibiae, tarsi, and claws) dorsally gold-silver with pink shine. Pronotum and head silver-gold. Clypeus and sometimes small rim around the eyes pink-brown, non reflecting due to fine puctures. Dorsal side of legs (femora, tibiae, and claws) shiny pinkbrown. Tarsi metallic gold-silver with a green shine. Ventral side of tibiae of all legs pinkbrown. Pygidium metallic silver-gold with green hue, shiny.

**Color variation:** A uniformly colored species based on material in the collection.

**Distribution in CNP:** Altitudinal range 1030–1147 m a.s.1. (Figure 3), recorded from Guanales only.

**Distribution:** Honduras and Guatemala (Thomas et al. 2006c).

**Remarks:** Identification based on male genitalia can be made through Morón (1990).

### Chrysina sp.1

**Size in CNP:** F 29.7–32.7 mm (n = 2).

**Recognition in the field:** This uniformly bright-green species most closely resembles *C. karschi*, but can be differentiated by the presence of faintly uncolored depressed rows on the elytra with numerous small punctures (Figure 6E). *Chrysina* sp.1 with hind tibiae proximally about twice as broad as distally and dorsally with 6–8 stout bristles, compared *C. karschi* with hind tibiae proximally about as broad as distally and with 2–4 minute bristles. Tarsi dark metallic brown-green (Figure 6–7E).

**Distribution in CNP:** Collected on a ridge close to Cortecito camp at an altitude of 1355 m a.s.1.

**Distribution:** Only known from CNP, Honduras.

**Remarks:** These two specimens differ from the CNP *Chrysina* species so far examined. They share similarities with *Chrysina auripes* Gray. Examination of the male genitalia is required to decide on species identity. Only two female specimen of *Chrysina* sp.1 were present in the collection, hence this species was not included in the analyses.

## Discussion

Out of the ten species listed for Honduras, six positively identified species and one hitherto unidentified species of jewel scarabs were recorded from CNP. This number amounts to over two-thirds of the national richness, which is remarkable for a small park such as CNP (about 200 km^2^). This result is not necessarily an indication for an exceptional high diversity in CNP or a poor *Chrysina* fauna in Honduras, but rather reflects the lack of information on Honduran *Chrysina* beetles. CNP is disproportionally well-studied for this genus compared to the remainder of the country. This lack of information is corroborated by the low number of species recorded from the central part of Central America, covered by the countries Honduras, El Salvador, Nicaragua, and Belize (Figure 2). These countries all have a low species count, but are surrounded by better surveyed countries with longer species lists, such as Mexico (48) and Guatemala (25) in the northern part of Central America and Costa Rica (19) and Panama (14) in the southern part.

The four other species recorded from Honduras are *C. bruyeai* (Hawks), *C. cavei* (Hawks and Bruyea), *C. ericsmithi* Monzon and Cano, and *C. luteomarginata* (Ohaus), and these can be reliably distinguished from the species recorded from CNP on the basis of male genital structure. *C. bruyeai* belongs to the “marginata group” (Hawks 1999) and is closest to *C. karschi*, but the genital capsule ends in three (*C. bruyeai*) versus two (*C. karschi*) processes. For *C. luteomarginata*, the male genital structure is asymmetrical, short, without metallic shine, with a left lateral wide and angulate processus (Morón 1990). *C. cavei* has strongly developed hind femora, which often have a dorsal bright orange-red coloration. *C. ericsmithi* is most similar to *C. pastori*, and can be distinguished by male genital structure (Monzon and Cano 1999).

All *Chrysina* species in CNP are easily separated based on color and external morphological characters. The most interesting specimens were the two unidentified female *Chrysina*, collected at mid-elevation on the mountain. These specimens have a superficial resemblance to *C. karschi* and might have been identified as such in previous surveys. This unidentified species only further indicates how, even in CNP, where light trapping has been ongoing since 2006 by Operation Wallacea, and much earlier by other researchers, the faunal assessment of *Chrysina* is incomplete and there is a need for further surveys. This situation is probably similar for many other parts of Honduras.

Some, mainly color-based, morphological variations were observed in the OUMNH collection. In all cases, conspecifity of these deviant morphs to the dominant form of the respective species was confirmed based on male genital structure. *C. karschi* is the only species in CNP that has a rare pink color morph. At least two other *Chrysina* species, *C. chrysargyrea* (Sallé) and *C. costata* (Blanchard), are known to have a similar pink color morph, but little is known on how common this variation is. More observations on the distribution and ecology of this pink morph would be valuable in order to reveal whether these are isolated specimens or established populations, and would help to gain insight into whether or not there are specific environmental factors that help sustain this pink morph. Other surprising and rare color forms include a green morph of *C. quetzalcoatli* and a *C. karschi* with an orange clypeus.

The distributional data presented for CNP are based on a small number of collection localities and only provide a preliminary insight into the ecology of the species under study. However, the patterns observed are clear, and the occurrences considered in the light of their respective elevations are in agreement with the observation that these *Chrysina* species typically are mountain-dwellers. This mountain-dwelling nature is further confirmed by the results of the potential distribution modeling. The two lowest camps, Santo Tomas (534 m a.s.1.) and the village of Buenos Aires (1144 m a.s.1.) have no records of *Chrysina*. Besides being the lowest in elevation, these two sites have well-established human populations, with most of the forest cleared. This deforestation obviously negatively affects the presence of *Chrysina*. If there were any *Chry-* *sina* beetles to be observed, *C. karschi* or *C. spectabilis* would be expected to be at these lower altitudes, based on their widespread recordings at most elevations (Figure 4) and the modeling. Species richness gradually increased with elevation, with the highest camp (Cantiles) containing all species except for *C. strasseni*. The morphologically very similar *C. pastori* and *C. strasseni* both belong to the “chrysargyrea group” (Hawks 2006) and seem to be elevationally separated. With only two collection localities of *C. strasseni*, this separation is difficult to quantify. Additional research is recommended to verify the extent to which both species co-occur. For now, *C. strasseni* appears to be the rarest species identified in CNP, with the smallest number of observations. It is the only species that was never found at high elevation.

The increasing richness of the *Chrysina* community with elevation contrasts with the classical and widely documented pattern of decreasing diversity with altitude (Hodkinson 2005). Exceptions to major richness patterns tend to occur in relatively small taxonomic groups, exemplified by the increase of Pinaceae towards the poles (Stevens and Enquist 1998) as contrasted to the generally observed decline on the latitudinal diversity gradient (Hillebrand 2004). The limited knowledge of the *Chrysina* species autoecology hampers understanding of the mechanisms behind the observed pattern, but the pattern clearly associates *Chrysina* species in CNP with undisturbed mountain forests and cloud forest habitat. More information about the diet specialization of both larval and adult stages would be highly valuable to understand the observed distribution patterns and evaluate the value of this group as cloud forest health indicator species.

*Chrysina* species are best sampled using light traps, of which there are numerous designs. However, as with any sampling methodology, standardization is the key in their use in ecological and monitoring studies. We suggest using a battery operated 25 W actinic light trap set against a white sheet. Since *Chrysina* are active flyers and may not stay at the light for more than a few minutes, we also suggest that the trap be manned throughout the trapping session to ensure that all specimens are sampled. To cover the activity peak of the different species, trapping sessions should run from just before dusk until midnight. When time is limited, the first two hours after dusk are advised. All specimens should have full data taken (e.g., species, sex, size, color morph, and time of appearance), and trapping should be performed for three consecutive nights. Ideally, population density estimates will be completed through capture-markrecapture studies (Southwood 1978). Habitat variables (forest type, elevation, topography) and environmental variables (temperature, humidity, rain) should be taken at each site during the trapping.

There is an acute need for an increased conservation effort of cloud forests (Aldrich et al. 2001). The use of charismatic groups as flagships can play a valuable role in engaging the broader public in the conservation of these unique habitats. These remarkable beetles can capture the imagination of the general public and foster their support of conservation. The clear association of *Chrysina* species with Neotropical mountain forests, along with their eye-catching habitus, makes jewel scarabs an ideal candidate group as ambassadors of the remaining Neotropical cloud forests.

## Abbreviations:

CNP,: Cusuco National Park;
OUMNH,: Oxford University Museum of Natural History
